# Decoding the Ektacytometric Landscape of Hereditary Spherocytosis: Insights from 204 Cases

**DOI:** 10.3390/diagnostics16142257

**Published:** 2026-07-20

**Authors:** Anna Zaninoni, Elisa Fermo, Cristina Vercellati, Anna Paola Marcello, Dario Consonni, Nayssen Naouara, Bruno Fattizzo, Wilma Barcellini, Paola Bianchi

**Affiliations:** 1 SC Ematologia, SS Fisiopatologia delle Anemie, Fondazione IRCCS Ca’ Granda Ospedale Maggiore Policlinico, Via Francesco Sforza, 35, 20122 Milan, Italy; anna.zaninoni@policlinico.mi.it (A.Z.); elisa.fermo@policlinico.mi.it (E.F.); cristina.vercellati@policlinico.mi.it (C.V.); anna.marcello@policlinico.mi.it (A.P.M.); nayssen.naouara@policlinico.mi.it (N.N.); bruno.fattizzo@policlinico.mi.it (B.F.); wilma.barcellini@policlinico.mi.it (W.B.); 2 SC Medicina del Lavoro, Fondazione IRCCS Ca’ Granda Ospedale Maggiore Policlinico, 20122 Milan, Italy; dario.consonni@policlinico.mi.it; 3 Dipartimento di Oncologia e Oncoematologia, Università degli Studi di Milano, 20122 Milan, Italy

**Keywords:** hereditary spherocytosis, rare anemia, ektacytometry

## Abstract

**Background**: Hereditary Spherocytosis (HS), the most common congenital hemolytic anemia, is characterized by highly heterogeneous clinical manifestations with variable hemolytic anemia, jaundice, reticulocytosis, splenomegaly, and gallstones. The ektacytometric analysis is considered a gold standard tool for HS diagnosis. Two distinct profiles may be observed: the typical bell-shaped profile (HS1) and a right-shifted curve (HS2); nonetheless, the clinical and biochemical significance of these differences, and their correlation with the clinical phenotype of HS remain poorly understood. **Methods**: We analyzed a large cohort of non-splenectomised HS patients, focusing on their ektacytometric curves and their hematologic and biochemical features. **Results**: We found that HS2 patients were significantly younger and showed a more severe anemia compared to HS1; in particular, HS2 patients had lower Hb levels and MCHC values, and higher RDW. Interestingly, only in the HS1 group, we observed a negative correlation between MCHC and all osmoscan parameters related to osmolality (Omin, Ohyper, and Omax). **Conclusions**: All the results indicate that HS may be characterized by two typical shapes of the ektacytometric curve, where HS2 profiles are associated with more severe and young patients, and that in the more HS severe cases red cells show a lower density.

## 1. Introduction

Hereditary hemolytic anemias constitute a heterogeneous group of rare disorders caused by defects in red blood cell (RBC) membrane structure and metabolism, leading to premature erythrocyte destruction or clearance. Clinically, these disorders are characterized by anemia of variable severity, splenomegaly, jaundice, and gallstones. Laboratory findings typically include abnormal RBC morphology, reticulocytosis, elevated unconjugated bilirubin and lactate dehydrogenase (LDH) levels, reduced haptoglobin, and frequently iron overload. Diagnostic evaluation relies on personal and family history, clinical assessment, and a tiered panel of laboratory investigations, some of which are available only in specialized centers. Overlapping clinical and hematologic features among different hereditary hemolytic anemias complicate differential diagnosis, particularly in mild or atypical phenotypes.

RBC membrane disorders result from qualitative or quantitative abnormalities of cytoskeletal proteins, which form a complex structure responsible for erythrocyte shape, deformability, hydration status, and volume regulation. These defects lead to characteristic morphological abnormalities detectable on peripheral blood smear. The most frequent membrane disorder is hereditary spherocytosis (HS), with a prevalence of approximately 1:2000–1:3000 in Caucasian populations. HS exhibits marked clinical heterogeneity, with manifestations including hemolytic anemia, jaundice, reticulocytosis, splenomegaly, and gallstones [1,2,3]. Inheritance is autosomal dominant in approximately 75% of cases, most commonly due to mutations in *ANK1*, *SLC4A1*, and *SPTB* genes, including de novo variants, whereas the remaining cases are autosomal recessive, often associated with biallelic defects in *SPTA1* or *EPB42* [4,5]. Protein deficiency or dysfunction leads to membrane surface area loss and the formation of osmotically fragile spherocytes with decreased deformability, ultimately resulting in premature splenic destruction [6,7].

Less common membrane disorders involve abnormalities in cation permeability and cell volume regulation, including dehydrated hereditary stomatocytosis (DHSt) caused by *PIEZO1* mutations, overhydrated hereditary stomatocytosis (OHSt) associated with *RhAG* mutations, and Gardos channelopathy due to *KCNN4* mutations. Rare stomatocytosis variants include pseudohyperkalemia and cryohydrocytosis with neurological impairment, caused by defects in *ABCB6* and *GLUT1*, respectively [3,4].

Hereditary hemolytic anemias may also arise from enzymatic deficiencies of erythrocyte metabolism or from defects in erythroid maturation, resulting in congenital dyserythropoiesis.

Given the overlapping clinical phenotypes, diagnosis requires a comprehensive diagnostic approach combining clinical data, hematologic testing (complete blood count, RBC indices, and hemolysis markers), and specialistic laboratory assays, including peripheral blood smear evaluation, osmotic fragility testing, eosin-5′-maleimide (EMA) binding test, ektacytometry, and membrane protein analysis [8,9,10]. Molecular testing using next-generation sequencing (NGS) is increasingly implemented to refine diagnosis; however, in the absence of supportive functional and clinical evidence, genetic findings alone are often insufficient to establish a definitive diagnosis [11]. NGS is particularly recommended when specialized functional assays are unavailable or following recent transfusions.

In the past years, ektacytometry has emerged as a reference technique for the diagnosis of HS and other RBC membrane disorders, including elliptocytosis, stomatocytosis, pyropoikilocytosis, and Southeast Asian ovalocytosis [12,13,14]. This technique simulates erythrocyte transit through microcirculation and, through osmotic gradient-induced deformation analysis, provides integrated information on RBC geometry, cytoplasmic viscosity, volume regulation, and membrane fluidity.

The original ektacytometer, limited by restricted availability, has largely been replaced by the Laser-assisted Optical Rotational Cell Analyzer (LoRRca MaxSis, Mechatronics Instruments BV^®^, Zwaag, The Netherlands), a robust and user-friendly platform, recently incorporated into diagnostic algorithms for RBC membrane disorders [12,13,15,16,17,18].

Osmoscan analysis enables sensitive detection of multiple cellular properties simultaneously. Key parameters include Omin, maximum elongation index (EImax), Ohyper, and area under the curve (AUC). Omin represents the osmolality at minimal deformability and corresponds to 50% RBC lysis in classical osmotic fragility tests, reflecting the mean surface-to-volume ratio. EImax reflects maximal deformability at isotonic osmolality and correlates with membrane surface area. Ohyper corresponds to the hypertonic osmolality at 50% of EImax and reflects cellular hydration status. AUC integrates deformability across the osmotic gradient.

Compared with normal RBCs, these parameters generate disease-specific signatures, enabling differential diagnosis of most membrane disorders. In HS, osmoscan curves typically show reduced EImax, increased Omin, and/or decreased Ohyper, resulting in a reduced AUC [12,13,15,16,17].

Two main HS ektacytometric phenotypes have been described by some authors. HS type 1 (HS1) is characterized by increased Omin, decreased Ohyper, and reduced EImax and AUC, whereas HS type 2 (HS2) exhibits a right-shifted curve with increased Omin and Ohyper and reduced EImax and AUC [17,18]. Although diagnostically informative, the clinical and biochemical correlation of these profiles, particularly their relationship with disease severity, remain incompletely defined. Recent studies have suggested associations between ektacytometric parameters and clinical severity. Novel osmoscan-derived biomarkers, including Omin-width, Omax-width, and physiological elongation indices, correlate with disease severity, red cell distribution width, and conventional hemolysis markers in retrospective HS cohorts. EImax and other curve-derived indices have also been associated with clinical severity and reticulocytosis, indicating a potential role for ektacytometry in severity stratification beyond diagnosis [18].

Overall, these observations support an evolving diagnostic paradigm in HS, integrating functional RBC phenotyping and comprehensive genotyping to refine diagnosis, elucidate pathophysiology, and inform personalized clinical management.

In this retrospective study, we analyzed a cohort of 204 non-splenectomized HS patients diagnosed at our center between 2014 and 2022, focusing on ektacytometric profiles and their correlation with hematological and biochemical parameters.

## 2. Materials and Methods

### 2.1. Patients

Peripheral blood was collected from patients during diagnostic procedures after obtaining informed consent and approval from the Institutional Human Research Committee. The procedures followed were in accordance with the Helsinki international ethical standards on human experimentation. The study was conducted according to the ICH Guideline for Good Clinical Practice. The study protocol was approved by the local ethical committee, Milano Area 2 as a sub-study of the observational and biologic study CYTOPAN (NCT05931718, approval date 3 May 2021). The great majority of samples were collected in our Institute; samples from other centers were shipped maintaining a temperature of 4 °C and processed within 24 h from sampling. All tests were performed in a single site using daily health controls as reference. None of the patients had been transfused within the three months preceding the study.

### 2.2. Diagnosis of Hereditary Spherocytosis

**Figure 1:**
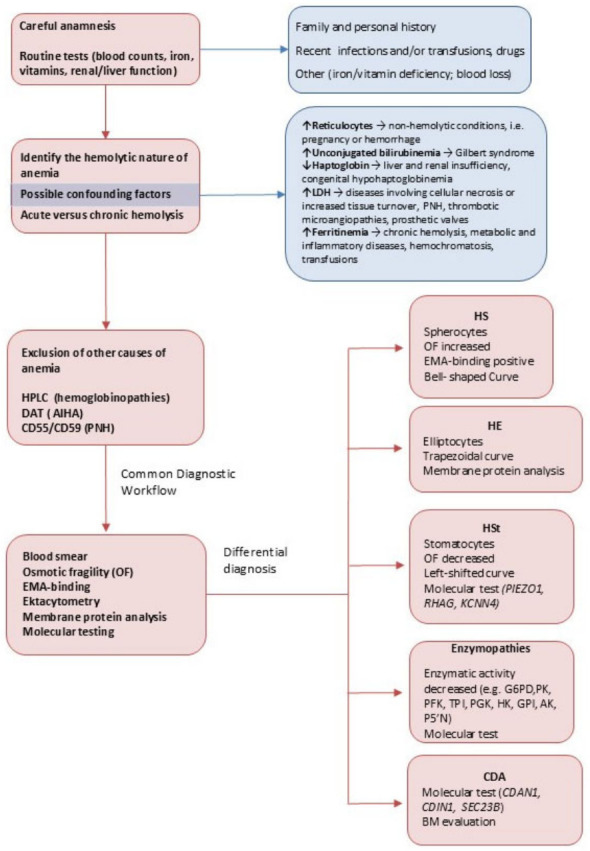
Diagnostic workflow and possible confounders in congenital hemolytic anemias (CHAs). DAT: direct antiglobulin test, HPLC: high performance liquid chromatography, EMA-binding: eosin-5′-maleimide-labeled RBC by flow cytometric analysis, HS: hereditary spherocytosis, HE: hereditary elliptocytosis, HSt: hereditary stomatocytosis, CDA: congenital dyserythropoietic anemia, G6PD: glucose-6-phosphate dehydrogenase, PK: pyruvate kinase, GPI: glucose phosphate isomerase, PFK: phospho-fructo-kinase, TPI: triose phosphate isomerase, PGK: phosphoglycerate kinase, HK: hexokinase, AK: adenylate kinase, P5N: pyrimidine 5′-nucleotidase deficiency. AIHA: autoimmune hemolytic anemia; PNH: paroxysmal nocturnal hemoglobinuria; BM: bone marrow.

Between 2014 and 2022, a total of 1450 patients with suspected membrane-related hemolytic anemia were referred to our Center. The diagnostic workflow for HS was performed according to the current guidelines [8,9,10,19] as reported in Figure 1.

Initial evaluation included clinical history and routine laboratory tests (complete blood count, iron status, vitamin levels, and liver and renal function), with assessment of potential contributing factors such as infections, transfusions, drugs, blood loss, and nutritional deficiencies. Hemolysis was defined based on increased reticulocyte count, elevated unconjugated bilirubin and LDH, reduced haptoglobin, and, in some cases, increased ferritin, while accounting for potential confounders. Alternative causes of anemia were excluded using high-performance liquid chromatography (HPLC), direct antiglobulin test (DAT), and CD55/CD59 analysis. The differential diagnosis of inherited hemolytic anemias was established through integrated morphological, biochemical, and molecular approaches. In particular, HS diagnosis was based on osmotic fragility tests (NaCl osmotic fragility test on both fresh and incubated blood, standard Glycerol Lysis test (GLT_50_), Acidified Glycerol Lysis test (AGLT_50_), Pink test [2], EMA-binding test [20], ektacytometric analysis by Laser-Assisted Optical Rotational Cell Analyzer (LoRRca) [12,13], and RBC membrane analysis by SDS-PAGE [2].

### 2.3. Ektacytometric Analysis

A 250 μL EDTA sample suspended in 5 mL of polyvinylpyrrolidone buffer (PVP, Mechatronics, Hoorn, The Netherlands) was used for the analysis. Osmoscan was performed by means of LoRRca according to the manufacturer’s instructions and as reported in detail by Zaninoni et al. (2018) [12]. The osmotic gradient curves reflect RBC deformability as a continuous function of suspending medium osmolality. The following parameters were evaluated: the Omin value corresponds to the osmolality at which the deformability reaches its minimum; the elongation index (EI) max corresponds to the maximal deformability or elongation obtained near the isotonic osmolality; the Ohyper (the osmolality in the hypertonic region corresponding to 50% of the EImax and the area under the curve (AUC), is defined in the provided software as the AUC beginning from a starting point in the hypo-osmolar region and an ending point in the hyper-osmolar region (instrument settings 500 mOsm/kg) [21].

Osmoscan curves were compared with the daily normal control and with the area covered from Osmoscan profiles obtained by healthy blood donors *(N* = 350). All the main parameters of the curve showed a Gaussian distribution, therefore the area covered by all the control curves was considered as the reference range.

### 2.4. Statistical Analysis

Wilcoxon rank-sum (also known as Mann–Whitney) and chi-squared tests were used to analyze quantitative and categorical variables, respectively. Spearman’s rho coefficient was used to assess correlations. Analyses were performed with Stata 19 (StataCorp, College Station, TX, USA, 2025).

## 3. Results

### 3.1. HS Patients

The diagnosis of HS was established based on the presence of anemia, a positive family history, clinical evidence of hemolysis, reduced fluorescence on the EMA-binding assay, increased osmotic fragility, an AGLT <900 s., and a characteristic ektacytometric profile [9,10].

Among the 1450 consecutive patients evaluated, 313 were ultimately diagnosed with HS. Of these, 204 (116 males and 88 females; median age 19 years, range 0.1–73), who had not undergone splenectomy and for whom complete clinical histories and datasets were available, were included in the study.

For the analysis, patients were stratified according to clinical severity and biochemical defects. HS patients were classified in asymptomatic (male Hb > 14 g/dL and female Hb > 12 g/dL), mild (Hb > 11 g/dL), moderate (Hb = 8–11 g/dL) and severe (Hb < 8 g/dL), according to Bolton-Maggs et al. [19], and successive guidelines [9,10]. Finally, based on SDS-PAGE analysis results, HS patients were stratified according to their membrane defects on the following groups: deficiency of band 3, spectrin, ankyrin, combined, or undetectable defect [2].

### 3.2. Ektacytometric Analysis

**Figure 2:**
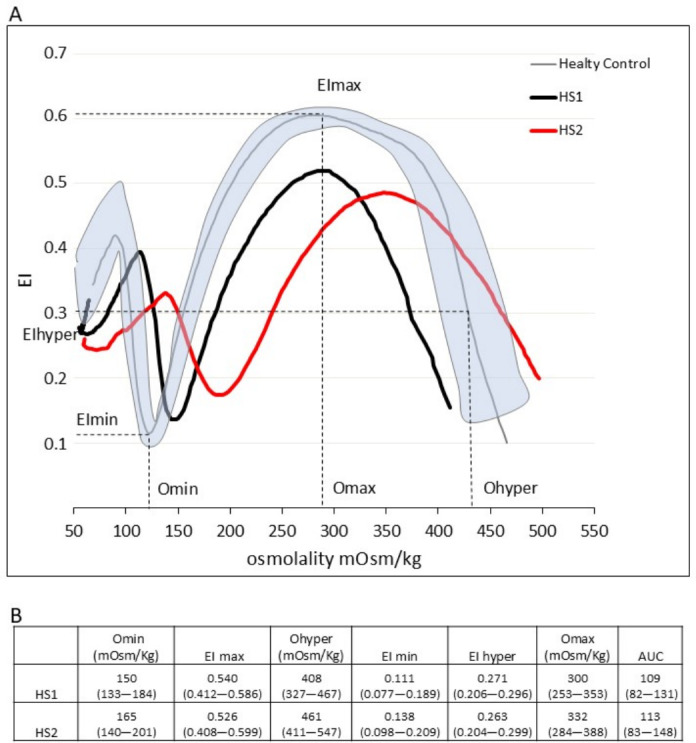
Ektacytometric profiles of hereditary spherocytosis (HS) patients. (**A**) Representative profiles of HS type 1 (HS1) and type 2 (HS2) patients compared to healthy controls. The blue shadow represents the area covered by healthy control profiles (N = 350). (**B**) Summary table reporting median (range) values of all the ektacytometric parameters in HS1 and HS2 subgroups to show within-group variability.

The ektacytometric profiles were obtained from all the 204 HS non-splenectomized patients by LoRRca Osmoscan analysis and we identified two distinct patterns: we defined HS1 profile as the curve with an Ohyper value lower than the daily control (median −7.02%, range −22.3 to −0.2; 153/204 patients), and HS2 profile as the curve with an Ohyper value higher than the daily control (median +4.3%, range +0.2 to +19.7; 51/204 cases) (Figure 2).

All laboratory and hematologic data were collected and analyzed together with Ektacytometric parameters. Comparing the two groups of patients (Table 1), we observed that HS2 patients were significantly younger than HS1 at the time of the study (10 years, range 0.1–71, versus 22 years, range 0.1–73, *p* = 0.002). Moreover, the HS2 group showed a higher number of severe cases and a lower number of mild cases compared to the HS1 group (13.7% versus 2.6% and 17.6% versus 36.6%, respectively, *p* = 0.004). In particular, in HS2 patients, Hb level and MCHC values were significantly lower, and RDW values were higher compared to HS1 (*p* = 0.004, *p* < 0.001, and *p* = 0.034, respectively). Although heterogeneous, no difference in median spherocyte number was observed in the two groups, nor in other atypical red cell shapes.

### 3.3. Laboratory Tests

Table 2 shows laboratory findings. We observed a greater number of HS2 patients with positive NaCl osmotic fragility test on fresh blood, (82.3% versus 60.1%, *p* = 0.007). No significant differences were found considering the other osmotic fragility tests, EMA-binding test, or RBC membrane protein defects, although spectrin deficiency was more frequent in HS2 patients contrary to band 3 defect that was more common in the HS1 group.

To further characterize HS1 and HS2 curves, we found other significant abnormalities in ektacytometric parameters (Table 3): as expected, ΔOmin median values were increased in both groups compared to daily controls although more markedly different in the HS2 group (*p* < 0.001), as also for ΔOmax median values (*p* < 0.001). Similarly, ΔEImin median values were more altered in HS2 compared to HS1 patients (28.8, range −3.4;113.3 versus 2.33, range −35.6;60.2, respectively, *p* < 0.001).

### 3.4. Correlation Analysis Between Ektacytometric Parameters and Laboratory and Hematologic Data

In the attempt to identify which parameter could influence the differences observed at ektacytometric analysis in the two groups, we analyzed the correlations between ektacytometric parameters and all laboratory and hematologic data. Although these analyses are exploratory and hypothesis-generating, several associations were revealed, as shown in Table 4. In both groups an association was observed between spherocytes and Omin, as well as between AGLT_50_ and EMA-binding with EImax, and with AUC; conversely, a negative correlation was observed between AGLT_50_, GLT_50_, Pink test, and Omin, and between spherocytes number with EImax. Interestingly, considering hematologic parameters, only in the HS1 group, MCHC showed negative correlations with osmolality-related osmoscan parameters (Omin *p* = 0.013, Ohyper *p* < 0.001, and Omax *p* = 0.003).

## 4. Discussion

Historically, the diagnosis of HS has always been considered a straightforward process, as it was commonly associated with the presence of hemolysis of variable degree, spherocytes, and decreased osmotic resistance. However, over the years, these cornerstone diagnostic parameters have proven to be insufficient. On the one hand, the marked clinical heterogeneity of the disease along with its overlap with other hemolytic conditions do not allow for a unique clinical classification. On the other hand, the number of spherocytes observed in the peripheral blood smear can vary widely and is often not reliably indicative of the diagnosis. Finally, although osmotic resistance tests were long regarded as the diagnostic gold standard, large case series have demonstrated their limited sensitivity—particularly in milder cases—and poor specificity, as positive results can also occur in other forms of congenital hemolytic anemia, frequently leading to misdiagnosis [20].

The EMA-binding test [22], along with its successive adaptations [23,24] and ektacytometry, nowadays represents one of the diagnostic methods with the highest sensitivity and specificity for HS diagnosis [25]. However, the EMA-binding test provides only a reduced fluorescence value, which indirectly reflects erythrocyte membrane instability due to a defect in one of cytoskeletal proteins involved. In contrast, the interpretation of ektacytometric curves yields extensive information regarding the erythrocyte defect and allows clinicians to raise diagnostic suspicion for various types of hemolytic anemia, not solely limited to hereditary spherocytosis. This makes the approach with ektacytometry particularly valuable, as it not only provides insights into the possible diagnosis but also allows to increase knowledge into pathophysiological aspects of defective red blood cells. Moreover, a more detailed interpretation of ektacytometric curves might provide additional information on anemia severity and prognosis.

Interestingly, in hereditary spherocytosis, an ektacytometric curve may display different shapes, although the underlying cause of this phenomenon is not yet fully understood [15,19]. In this study, we investigated these two ektacytometric profiles (HS1 and HS2) in a large cohort of non-splenectomized patients with hereditary spherocytosis, classifying them according to the relative position of the Ohyper parameter compared with daily controls. Splenectomized patients were excluded from the analysis to avoid potential bias in the interpretation of the results; in fact, in these patients, older and morphologically abnormal red blood cells persist in circulation because they are no longer removed by the spleen, leading to ektacytometric profiles that differ from those observed in non-splenectomized individuals [12].

Notable differences were observed between HS1 and HS2 groups. Patients displaying the HS2 profile were significantly younger and showed a higher proportion of severe cases; they also showed lower hemoglobin and MCHC levels, along with higher RDW values. These findings suggest that the HS2 profile may be associated with a more pronounced impairment of red blood cell deformability and increased clinical severity. This interpretation is consistent with studies demonstrating that a reduced elongation index and area under the curve correlate with anemia severity and hemolytic burden in hereditary spherocytosis [26]. Moreover, HS2 patients more frequently had positive NaCl osmotic fragility tests in fresh blood, further supporting the hypothesis of increased membrane instability in this subgroup. Osmotic fragility has long been linked to the loss of surface-to-volume ratio and decreased RBC deformability in HS, and its association with altered ektacytometric parameters has been extensively documented [27]. Although no statistically significant differences were observed between the HS1 and HS2 groups regarding EMA-binding test positivity or overall membrane protein defects, some trends emerged: spectrin deficiency was more frequently detected in HS2 patients, whereas band 3 defects predominated in HS1. This pattern is consistent with previous observations suggesting that spectrin-related defects often lead to more severe cytoskeletal instability and reduced membrane resilience, although genotype–phenotype correlations in hereditary spherocytosis remain complex and heterogeneous [13,28,29].

In our cohort, both HS1 and HS2 groups showed increased ∆Omin and ∆Omax values compared with daily controls, indicating a reduced surface-to-volume ratio and altered cellular hydration. These alterations were more pronounced in HS2 patients, who also exhibited a greater reduction of ∆EImin. Increased Omin values and reduced elongation indices are well-established markers of impaired red blood cell deformability and have been consistently associated with disease severity in hereditary spherocytosis [26,27].

Correlation analysis further demonstrated a positive association between the number of spherocyte and Omin, as well as a negative correlation between spherocyte burden and EImax. These findings support the relationship between spherocytosis, reduced red blood cell deformability, and increased osmotic fragility [26].

Interestingly, a negative correlation between MCHC and osmolality-related osmoscan parameters (Omin, Ohyper, and Omax) was observed only in the HS1 group. Elevated MCHC is classically associated with membrane surface loss and cellular dehydration in HS; however, previous studies have reported inconsistent relationships between MCHC and deformability indices, likely due to the influence of cellular heterogeneity and compensatory erythropoietic mechanisms [26,30]. The absence of this correlation in HS2 patients suggests that, in this subgroup, more evident cytoskeletal disruption or altered membrane hydration may play a dominant role in determining the ektacytometric profile. However, since multiple correlations were examined and no adjustment for multiple testing was applied, this result should be considered hypothesis-generating and warrants validation in independent patient cohorts and through integrated molecular, proteomic, and functional studies.

Given the marked molecular heterogeneity underlying hereditary spherocytosis and the large number of different genes involved in its pathogenesis, we chose not to include molecular defects in our analysis. This decision was also influenced by the fact that genetic characterization was not available for all patients and is not currently required by diagnostic guidelines. For example, defects in *SLC4A1* can lead to different phenotypic expressions depending on the mutation site. Variants may result in classical hereditary spherocytosis, in sphero-stomatocytosis when mutations affect residues involved in the protein’s transport activity, or in Southeast Asian Ovalocytosis when the defect occurs in the transition region between the cytoplasmic and transmembrane domains of the protein. Similarly, *SPTA1* defects— that together with band 3 abnormalities are among the most common causes of HS—are typically inherited in an autosomal recessive manner. However, they may also result from the combination of a pathogenic *SPTA1* allele inherited *in trans* with a low-expression polymorphic allele (e.g., *SPTA1* αLely or *SPTA1* αLePra). In all these conditions, the ektacytometric curve exhibits distinct features that could potentially confound the stratification and correlation analysis between HS1 and HS2 profiles. Although a detailed analysis of correlation between molecular defects and ektacytometric parameters has recently been performed in a large cohort of HS patients [31], no clear distinction between HS1 and HS2 ektacytometric patterns was identified.

Overall, the results of this study support the concept that ektacytometric analysis provides not only diagnostic information but also valuable insights into the functional and clinical heterogeneity of hereditary spherocytosis. The identification of two distinct osmoscan profiles, associated with different hematological features and severity patterns, further highlights the role of ektacytometry as a key tool for patient stratification and disease assessment of disease severity [12,13,26,32,33]. However, this is a cross-sectional study, and longitudinal follow-up data were not available. Consequently, we cannot determine whether HS1 and HS2 profiles remain stable over time, evolve during the natural history of the disease, or predict subsequent clinical outcomes and treatment decisions. Prospective longitudinal studies incorporating repeated ektacytometric assessments will be required to address these questions.

## Figures and Tables

**Table 1:** Demographics, hematologic, and biochemical data of 204 patients with hereditary spherocytosis (HS) divided according to osmoscan profile (HS1, HS2).

	HS1 (*N* = 153)	HS2 (*N* = 51)	*p* Values *
Age (year)	22 (0.1–73)	10 (0.1–71)	*p* = 0.002
Gender M/F	93/60	23/28	NS
Type of anemia N (%)			*p* = 0.004
compensated	49 (32)	18 (35.3)	
mild	56 (36.6)	9 (17.6)	
moderate	44 (28.8)	17 (33.4)	
severe	4 (2.6)	7 (13.7)	
Hb (gr/dL)	12 (6.3–16.4)	11.1 (7.0–15.1)	*p* = 0.004
MCV (fL)	83.4 (68.4–98.7)	83.2 (59.6–101.0)	NS
MCHC (gr/dL)	36.1 (24.9–40.1)	34.5 (29.3–39.1)	*p* < 0.001
RDW (%)	17.8 (13.2–26.7)	19.1 (12.2–27.9)	*p* = 0.034
Reticulocytes count (10^9^/L)	286 (18–781)	300 (35–700)	NS
Unconjugated bilirubin (mg/dL)	2.0 (0.2–16.3)	2.0 (0.1–9.6)	NS
LDH (IU/mL)	259 (133–543)	255 (144–903)	NS
Iron (μg/dL)	81 (40–236)	90 (25–168)	NS
Transferrin (mg/dL)	218 (148–336)	230 (150–337)	NS
Transferrin saturation (%)	27 (14–85)	34 (13–46)	NS
Ferritin (ng/mL)	250 (28–1180)	198 (16–629)	NS
Spherocytes (%)	9 (1–44)	9 (1–59)	NS

Values are expressed as median (min–max) or N (%) as appropriate. Hb: Hemoglobin; MCV: Mean Corpuscular Volume; MCHC: Mean Corpuscular Hemoglobin Concentration; RDW: Red Cell Distribution Width; LDH: Lactate dehydrogenase; Normal ranges: Hb 13.6–16.7 gr/dL. MCV 80–96 fL, MCHC 32–36 gr/dL, RDW 11.8–15.6%, Reticulocytes count 16–84 10^9^/L, Unconjugated Bilirubin <0.76 mg/dL, LDH 122–222 IU/mL, Iron 50–176 μg/dL, Transferrin 240–360 mg/dL, Transferrin saturation 15–50%, Ferritin 15–150 ng/mL. * Calculated with Wilcoxon rank-sum test (quantitative variables) or chi-squared test (categorical variables).

**Table 2:** Laboratory data of 204 patients with hereditary spherocytosis (HS) divided according to osmoscan profile (HS1, HS2).

	HS1 (*N* = 153)	HS2 (*N* = 51)	*p* Values *
Positive HS screening tests N (%)			
GLT_50_	106 (69.3)	37 (72.5)	NS
AGLT_50_	146 (95.4)	49 (96)	NS
OF NaCl on fresh blood	92 (60.1)	42 (82.3)	*p* = 0.007
OF NaCl on incubated blood	121 (79)	45 (88.2)	NS
Pink test	140 (91.5)	50 (98)	NS
EMA-binding test	150 (98)	48 (96)	NS
RBC membrane defects N (%)			NS
Band 3	72 (47.1)	16 (31.4)	
Spectrin	45 (29.4)	20 (39.2)	
Ankyrin	1 (0.7)	0 (0)	
Combined defect ^§^	8 (5.2)	3 (5.9)	
Undetected	27 (17.6)	12 (23.5)	

Values are expressed as N (%). GLT_50_: Standard Glycerol Lysis test; AGLT_50_: Acidified Glycerol Lysis test; OF: osmotic fragility; EMA: eosin-5′-maleimide. The cut offs for considering positive test were: GLT_50_ hemolysis in <23 s, AGLT_50_ hemolysis in <900 s, decreased OF NaCl on fresh and incubated blood, Pink test hemolysis >33%, EMA-binding test fluorescence reduction <11%. ^§^ Combined defect: Band 3 + Band 4.2, Spectrin + Band 4.2; Spectrin + Ankyrin, Band 3 + Ankyrin. * Calculated chi-squared test.

**Table 3:** Percentage of variation of osmoscan parameters, compared to control, in 204 patients with hereditary spherocytosis (HS) divided according to osmoscan profile (HS1, HS2).

Ektacytometric Parameters	HS1 (*N* = 153)	HS2 (*N* = 51)	*p* Values *
Omin (mOsm/Kg)	17.6 (−2.8;35.9)	26.3 (−4.4;47.7)	*p* < 0.001
EI max	−11.9 (−29.6;−2.8)	−14.1 (−32.3;−1.0)	*p* = 0.03
EI min	2.33 (−35.6; 60.2)	28.8 (−3.4;113.3)	*p* < 0.001
EI hyper	−11.6 (−29.7;−2.3)	−14.0 (−32.2;−1.0)	*p* = 0.02
Omax (mOsm/Kg)	6.9 (−8.7;22.9)	14.2 (−8.45;29.1)	*p* < 0.001
AUC	−28.3 (−77.7; −10.4)	−24.02 (−43.2;6.65)	*p* = 0.006

Values are expressed as median percentage of variation (range) compared to daily normal control. EI: Elongation Index; O: Osmolality; AUC = Area under the curve. * Calculated with Wilcoxon rank-sum test.

**Table 4:** Spearman’s rho correlation coefficients between ektacytometric parameters and main laboratory and hematologic data in patients with hereditary spherocytosis (HS) stratified according to osmoscan profile (HS1, HS2).

HS1 (*N* = 153)	Omin (mOsm/Kg)	EI Max (N)	Ohyper (mOsm/Kg)	Omax (mOsm/Kg)	AUC (N)
Hb (g/dL)	−0.06	0.11	−0.11	0.04	−0.06
MCV (fL)	0.04	0.14	−0.02	0.07	−0.01
MCHC (g/L)	−0.20 *	0.01	−0.32 *	−0.28 *	−0.21 *
Spherocytes (%)	0.21 *	−0.54 **	0.07	0.02	−0.42 **
Reticulocytes Count (10^9^/L)	0.11	−0.51 **	−0.08	−0.15 *	−0.42 **
EMA-binding (%)	−0.24 *	0.50 **	−0.00	0.00	0.49 **
**HS2 (*N* = 51)**					
Hb (g/dL)	0.18	0.21	0.08	0.23	0.06
MCV (fL)	0.20	−0.06	0.16	0.37 *	−0.10
MCHC (g/L)	0.20	−0.36 *	−0.05	0.09	−0.42 *
Spherocytes (%)	0.36 *	−0.71 **	0.00	0.16	−0.66 **
Reticulocytes Count (10^9^/L)	0.42 *	−0.65 **	0.08	0.27	−0.65 **
EMA-binding (%)	0.21	0.66 **	0.11	−0.03	0.63 **

EI: Elongation Index; O: Osmolality; AUC: Area under the curve; Hb; Hemoglobin; MCV: Mean Corpuscular Volume; MCHC: Mean Corpuscular Hemoglobin Concentration; EMA: eosin-5-maleimide. * *p* <0.05; ** *p* <0.001.

## Data Availability

The original contributions presented in this study are included in the article. Further inquiries can be directed to the corresponding author.
